# Development and Aging of the Kisspeptin–GPR54 System in the Mammalian Brain: What are the Impacts on Female Reproductive Function?

**DOI:** 10.3389/fendo.2013.00022

**Published:** 2013-03-28

**Authors:** Isabelle Franceschini, Elodie Desroziers

**Affiliations:** ^1^UMR85 Physiologie de la Reproduction et des Comportements, Institut National de Recherche AgronomiqueNouzilly, France; ^2^UMR7247, Centre National de la Recherche ScientifiqueNouzilly, France; ^3^Université François Rabelais de ToursTours, France; ^4^Institut Français du Cheval et de l’EquitationNouzilly, France

**Keywords:** kisspeptin, GPR54, ontogenesis, neuron, differentiation, regulation, reproduction, environment

## Abstract

The prominent role of the G protein coupled receptor GPR54 and its peptide ligand kisspeptin in the progression of puberty has been extensively documented in many mammalian species including humans. Kisspeptins are very potent gonadotropin-releasing hormone secretagogues produced by two main populations of neurons located in two ventral forebrain regions, the preoptic area and the arcuate nucleus. Within the last 2 years a substantial amount of data has accumulated concerning the development of these neuronal populations and their timely regulation by central and peripheral factors during fetal, neonatal, and peripubertal stages of development. This review focuses on the development of the kisspeptin–GPR54 system in the brain of female mice, rats, sheep, monkeys, and humans. We will also discuss the notion that this system represents a major target through which signals from the environment early in life can reprogram reproductive function.

## Introduction

The G protein coupled receptor GPR54 was initially cloned based on its high homology with the galanin receptor (Lee et al., 1999). Its peptide ligands were purified from human placenta (Ohtaki et al., 2001) and identified as the proteolysis products of a 145 amino acid protein encoded by the tumor suppressor gene *Kiss1* (Lee et al., 1996). These ligands have been termed kisspeptins and share a 10 amino acid long sequence at their amidated C terminal, a sequence that is sufficient to bind and activate GPR54 (Ohtaki et al., 2001). The importance of the kisspeptin–GPR54 system in reproductive function came to light in 2003 when it was discovered that some human families displaying hypogonadotropic hypogonadism and absence or delay in puberty bore mutations in the *GPR54* gene (de Roux et al., 2003; Seminara et al., 2003).

**Figure 1:**
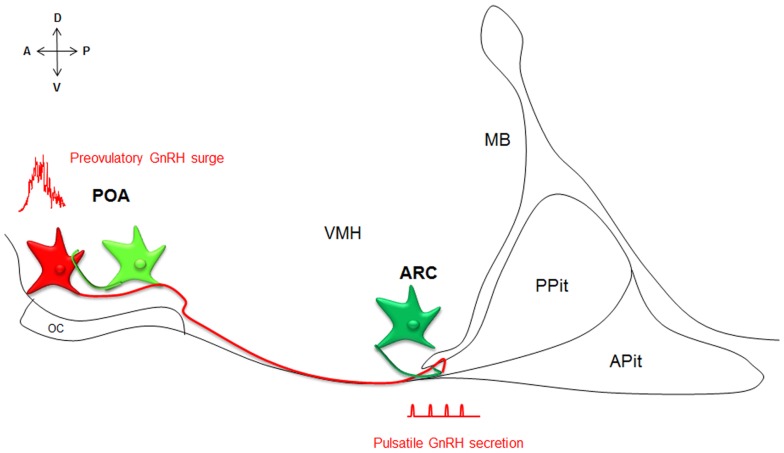
**Neuroanatomy of the kisspeptin–GPR54 system**. Simplified scheme of a midsagittal section through the ventral forebrain, representing the neuroanatomy of the kisspeptin–GPR54 system. Two anatomically and phenotypically distinct populations of kisspeptin neurons (green) control GnRH (red) secretion: kisspeptin neurons in the POA drive GnRH surges and kisspeptin cells in the ARC modulate the tonic pulsatile release of GnRH. Both populations of kisspeptin neurons interact with GnRH neurons in part directly. A, anterior; P, posterior; D, dorsal; V, ventral; POA, preoptic area; ARC, arcuate nucleus; OC, optic chiasm, VMH, ventromedial hypothalamus; MB, mammillary bodies; APit, anterior pituitary; PPit, posterior pituitary.

*Kiss1* and *GPR54* have since been cloned from many mammalian species. Kisspeptin–GPR54 signaling predominantly acts at the level of the brain to control reproductive function (Oakley et al., 2009 for review). Kisspeptins are in fact the most potent gonadotropin-releasing hormone (GnRH) peptide secretagogue discovered to date (Messager et al., 2005). Kisspeptins are produced by two main populations of neurons, one within the preoptic area (POA) and one within the arcuate nucleus (ARC, or infundibulum in primates), that have been implicated in the regulations of the preovulatory GnRH surge and GnRH pulsatile release, respectively (Figure 1; Lehman et al., 2010 for review). Importantly, kisspeptin neurons express a large variety of hormonal receptors not expressed by GnRH neurons and may integrate and convey to GnRH neurons a large panel of information about the body, enabling adaptive outcomes on reproduction.

Within the last 2 years, a substantial amount of data has accumulated on the physiological regulation and function of the kisspeptin–GPR54 system in the developing brain and we consider it as timely and worthwhile to review this topic. In addition, we review recent evidence indicating that the kisspeptin–GPR54 system may represent a major target through which signals from the environment early in life can produce long-lasting defects on reproductive function. This review focuses on females and discusses data from mice, rats, sheep, monkeys, and humans in separate sections since marked differences exist between species and sexes relative to the developmental regulation of the kisspeptin–GPR54 system. For more detailed specific information on the neuroanatomy and regulation of the system in developing males or in adulthood, please refer to recent reviews on these subjects (Lehman et al., 2010; Clarke, 2011; Poling and Kauffman, 2013). Barely a decade has passed since the discovery of the importance of GPR54 in the development of reproductive function and, despite the relative youth of the field, some consensus ideas and matters of controversies are starting to emerge with implications for important future directions.

## A Role for the Kisspeptin–GPR54 System in Puberty and Sexual Differentiation

The prominent involvement of central nervous system kisspeptin signaling in the maturation of reproductive function is strongly suggested from genetic association studies of developmental reproductive disorders in humans and from experimental data on a variety of animal models. These studies are detailed below for each species and summarized in Table 1.

**Table 1:** **Summary of studies showing a role of kisspeptin–GPR54 system in the development of female reproductive function**.

Approaches	Species	Reference	Manipulation	Period of manipulation	Time of analysis	Physiological impact	Hypothalamic analysis
Genetic	Human	Seminara et al. (2003), de Roux et al. (2003), Nimri et al. (2011)	GPR54 mutations (loss of function)	Congenital	Adult	IHH	
		Teles et al. (2008)	GPR54 mutations (gain of function)		Child	ICPP	
		Silveira et al. (2010)	Kiss1 mutations	Congenital	Child	IHH and ICPP	
		Topaloglu et al. (2012)			Child and Adult	IHH	
	Mouse	Seminara et al. (2003)	GPR54-KO	Congenital	Peripubertal to adult	HH, puberty delayed, no estrus cycle	No change in GnRH content (RIA)
		Funes et al. (2003)			Peripubertal to adult	HH, infertility	
		Lapatto et al. (2007)			Peripubertal to adult	HH, puberty delayed, no estrus cycle	
		Dungan et al. (2007)			Peripubertal to adult	HH, low circulating LH even after ovariectomy	No change in GnRH cell numbers
		Clarkson et al. (2008)			Peripubertal to adult	Compromised ability to mount LH surges	
		Lapatto et al. (2007)	Kiss1-KO	Congenital	Peripubertal to adult	HH, puberty delayed, no estrus cycle	
		d’Anglemont de Tassigny et al. (2007)			Peripubertal to adult	HH, puberty delayed, low circulating LH levels	No change in GnRH content (RIA), GnRH cell numbers and median eminence fiber density
		Mayer and Boehm (2011)	Kiss1 cell ablation Kiss1-DTA	Congenital	Prepubertal (P20) and adult	HH, estrus cycle present but less regular than control	Decrease in Kiss1 mRNA in ARC and kisspeptin-immunoreactive cell numbers in POA
		Kiss1 conditional cell ablation	Prepubertal (P20)	Pubertal (P40) and adult	No estrus cycle: persistent diestrous, infertility	
Pharmacological	Rat	Matsui et al. (2004)	Kp s.c. injection (acute)	Prepubertal (P25)	Prepubertal (P25)	Increase in circulating LH	Effect abolished by GnRH receptor antagonist
		Navarro et al. (2004b)	Kp i.c.v. injection (acute)	Prepubertal (P25)	Prepubertal (P25)	Increase in circulating LH, decrease in circulating prolactin	
		Navarro et al. (2004a)	Kp i.c.v. injections (chronic)	Prepubertal (P26–P31)	Prepubertal (P26–P31)	Puberty advanced, increase of uterus weight, increase in circulating LH and E2	LH-releasing effect abolished by GnRH receptor antagonist
		Castellano et al. (2006)	Kp i.p. injection (acute)	Neonatal (P5), Prepubertal (P15 and P25)	Neonatal (P5), Prepubertal (P15 and P25)	Increase in circulating LH but significant only at P15	
		Pineda et al. (2010)	GPR54-antagonist i.c.v. infusion	Prepubertal (from P30 to P36)	Prepubertal (P36)	Puberty delayed	
	Sheep	Redmond et al. (2011a)	Kp i.v. injection	Prepubertal (28 W)	Prepubertal (28 W)	Increase in LH pulsatility (frequency and amplitude) and circulating E2	
	Monkey	Guerriero et al. (2012a)	Kp i.c.v. injection	Prepubertal (52–81 W) and pubertal (112–175 W)	Prepubertal (52–81 W) and pubertal (112–175 W)		Increase in GnRH pulsatile release GNRH-response to kp increases at puberty
			GPR54-antagonist i.c.v. injection	Prepubertal (52–81 W) and pubertal (112–175 W)	Prepubertal (52–81 W) and pubertal (112–175 W)		Decrease in GnRH pulsatile release only at prepubertal stage
		Roseweir et al. (2009)	GPR54-antagonist i.c.v. injection	Pubertal (132 W)	Pubertal (132 W)		Decrease in GnRH pulsatile release and basal GnRH (microdialysis)

*IHH, idiopathic hypogonadotropic hypogonadism; ICPP, idiopathic central precocious puberty; HH, hypogonadotropic hypogonadism; s.c., sub cutaneous; i.v., intravenous; i.p., intraperitoneal; i.c.v., intracerebroventricular; RIA, radioimmunoassay; POA, preoptic area; ARC, arcuate nucleus; ME, median eminence; GnRH, gonadotropin-releasing hormone; LH, luteinizing hormone; E2, estradiol; P, postnatal day; W, weeks*.

### Mouse

In the mouse, targeted genetic disruption of *GPR54* (de Roux et al., 2003; Funes et al., 2003; Seminara et al., 2003) or of *Kiss1* (d’Anglemont de Tassigny et al., 2007; Lapatto et al., 2007) produce a similar hypogonadotropic hypogonadism phenotype, suggesting that kisspeptins represent the main ligands for this receptor. This major effect on the development of reproductive function does not appear to involve a reduction of GnRH peptide levels in the brain nor a reduction in GnRH neuronal numbers (Seminara et al., 2003; d’Anglemont de Tassigny et al., 2007; Lapatto et al., 2007) but involves an impairment in tonic GnRH release (Dungan et al., 2007). *In vitro* studies further suggest that central kisspeptins can already signal developing GnRH neurons well before birth. Nasal placodes from embryonic day (E) 11.5 mouse embryos can be explanted *in vitro* to produce a GnRH neuronal network that releases GnRH in a pulsatile manner (Constantin et al., 2009a). If kisspeptins are applied to these cultures, it increases both GnRH pulse frequency and pulse amplitude (Constantin et al., 2009a). Kisspeptins have also been shown to promote neurite outgrowth of GnRH neurons from embryonic POA explants, suggesting their participation in morphogenetic events (Fiorini and Jasoni, 2010). Notably, developmental strategies leading to the maturation of the GnRH system through puberty appear remarkably plastic at early stages of mouse development. For instance, if kisspeptin neurons are conditionally ablated with a toxin during the juvenile stage, mice display hypogonadism, persistent diestrus, and infertility but this is not the case if these cells are congenitally ablated indicating that strong compensatory mechanisms can occur earlier in development (Mayer and Boehm, 2011). *GPR54* deletion has also been found to inhibit defeminization/masculinization of brain circuits and behavior in males (Kauffman et al., 2007a) without interfering with the perinatal testosterone surge (Poling and Kauffman, 2012). In particular, *GPR54* knock-out males display a pattern of kisspeptin expression in the POA and an olfactory-mediated partner preference behavior characteristic of females (Kauffman et al., 2007a). By contrast, no behavioral deficits have yet been reported in female *GPR54* knock-out mice (Kauffman et al., 2007a). Nevertheless, a major neuroendocrine effect has been found since these females become unable to perform a surge of luteinizing hormone (LH) under estrogen positive feedback conditions in adulthood (Clarkson et al., 2008), raising the possibility that the development of female-specific brain circuits may be impacted by this genetic deletion. Further investigations on the impact of the kisspeptin–GPR54 system on the sexual differentiation of the brain and behaviors in females remain an important future direction.

### Rat

The involvement of the kisspeptin–GPR54 system in the central maturation of rat reproductive function has been explored with great precision by the group of Tena-Sempere since 2004 (Navarro et al., 2004a,b; Castellano et al., 2005, 2006). These studies used various modes of administration of synthetic forms of kisspeptins at different stages of development, followed by the monitoring of endocrine and physiological changes. In prepubertal female rats, kisspeptins, whether administered centrally or peripherally, induce an immediate elevation of circulating LH levels and this effect can be abolished by central administration of a GnRH receptor antagonist (Matsui et al., 2004; Navarro et al., 2004a). The GnRH-releasing activity of kisspeptins was directly tested on hypothalamic explants derived from female rats of various postnatal stages and found similar between the neonatal, infantile, and juvenile stages (Castellano et al., 2006). Repeated intracerebroventricular injections during the late juvenile period are able to increase uterus weight, circulating levels of LH and estradiol, and to advance the age of vaginal opening, a peripheral landmark of puberty onset in rodents (Navarro et al., 2004a). Conversely, central infusion of a GPR54 antagonist to peripubertal female rats decreases uterus and ovary weights without affecting total body weights and delays puberty onset (Pineda et al., 2010). Taken together, these observations provide convincing evidence that prepubertal kisspeptins are both necessary and sufficient for triggering various indices of female puberty in this species.

### Sheep

The functional relevance of the kisspeptin–GPR54 system in the maturation of female reproductive function has been well characterized during the last decade in mice and rats, as detailed in the preceding sections; however it was not until 2011 that this aspect has been explored in species other than rodents such as sheep and monkeys. In ewes, chronic hourly intravenous injections of kisspeptins during the prepubertal period stimulated a pulsatile release of LH within 15 min following injections, increasing both pulse frequencies and amplitudes (Redmond et al., 2011a). Mean circulating levels of LH and estradiol were increased and a surge like release of LH developed in some lambs 17 h post treatment. These animals were however unable to develop a long-lasting luteal phase as attested by circulating progesterone levels and did not achieve regular estrus cyclicity, suggesting that the reproductive neuroendocrine axis was not yet fully mature (Redmond et al., 2011a).

### Monkey

In female monkeys, it has recently become possible to directly monitor the GnRH-releasing activity of kisspeptins *in vivo* (Guerriero et al., 2012a). The GnRH-releasing response to kisspeptin infusion directly within the medial basal hypothalamus and stalk median eminence is dose dependent and greater in pubertal than prepubertal females (Guerriero et al., 2012a). Conversely, mean GnRH levels are diminished following central infusion with a GPR54 antagonist at both developmental stages (Roseweir et al., 2009; Guerriero et al., 2012a). This provides convincing evidence that a GPR54-mediated mechanism is required for the reactivation of GnRH release at puberty in the female monkey.

### Human

The fundamental role of the kisspeptin–GPR54 system in Pubertal development was initially discovered by two independent groups that identified loss of function mutations in the *GPR54* gene within human families with idiopathic hypogonadotropic hypogonadism (IHH) (de Roux et al., 2003; Seminara et al., 2003). Mutations of the *GPR54* gene have since been found in other families with IHH associated with various levels of GnRH deficiency (Nimri et al., 2011; Wahab et al., 2011). A very recent report also discovered an inactivating mutation of the *Kiss1* gene associated with IHH (Topaloglu et al., 2012). Idiopathic central precocious puberty (ICPP) is another developmental reproductive disorder that has been associated with either *GPR54* mutations (Teles et al., 2008), *Kiss1* mutations (Silveira et al., 2010), or polymorphisms (Luan et al., 2007a,b). Taken together, these pathologies attest for the critical role played by kisspeptin signaling in the development of the human hypothalamic–pituitary–gonadal axis.

## Developmental Changes of Kisspeptin–GPR54 Coincide with Changes in LH Secretion Throughout Life

### Mouse

Recent evidence points to an early onset of *Kiss1* and *GPR54* expression in the mouse nervous system. For instance, *Kiss1* mRNA has been detected in the mediobasal hypothalamus of the mouse as early as embryonic day (E)13, by reverse transcription polymerase chain reaction (Fiorini and Jasoni, 2010). At this early developmental stage, *in situ* hybridization further identified *GPR54* mRNA in some GnRH neurons along the nasal portion of their migratory route (Constantin et al., 2009b). Furthermore, *in vitro* studies suggest that GnRH neurons are already able to respond to kisspeptins by enhanced secretion during prenatal life (Constantin et al., 2009a,b). However, the presence of the kisspeptin protein remains to be shown in mouse embryos.

Postnatally, *Kiss1*-expressing cells can already be detected in the ARC a few hours only after birth, using *in situ* hybridization (Poling and Kauffman, 2012). *Kiss1* mRNA levels increase in this region at the time of puberty but only in hypogonadal hpg mice, which are deficient in GnRH receptor signaling and hence display very low levels of sex steroids (Gill et al., 2010, 2012). In wild type mice, it appears that *Kiss1* expression in the ARC is strongly repressed postnatally by circulating estradiol and no developmental changes have yet been detected in this region at the *Kiss1* transcript or kisspeptin protein level (Clarkson and Herbison, 2006; Gill et al., 2010, 2012). Of interest, however, is the significant increase in neurokinin B (NKB; another GnRH secretagogue expressed by kisspeptin neurons) transcript levels that has been observed in this nucleus prior to puberty onset (Gill et al., 2012). In the POA, expression of *Kiss1* appears to develop later than in the ARC, between postnatal day 8 and 10 (Semaan et al., 2010) and increases thereafter until puberty is reached (Gill et al., 2010). Similarly, a peripubertal increase in the number of POA kisspeptin-immunoreactive neurons has been shown (Clarkson and Herbison, 2006; Clarkson et al., 2009; Gill et al., 2010; Mayer et al., 2010). This increase has been correlated with the development of the capacity for GnRH/LH surges (Clarkson et al., 2009). Another interesting feature observed in the development of this system is a peripubertal increase in the proportion of GnRH neurons closely apposed to kisspeptin-immunoreactive fibers, suggesting a postnatal morphological maturation of kisspeptin circuitry associated with puberty (Clarkson and Herbison, 2006). Furthermore, a longitudinal analysis of transgenic mice in which *LacZ* has been introduced in the *GPR54* locus identified a prepubertal rise in the percentage of GnRH neurons expressing *GPR54* (Herbison et al., 2010). These different changes observed in the female POA at the time of puberty may be related to the increase in circulating LH levels that precedes puberty onset in the mouse (Michael et al., 1980; Gill et al., 2012). In any case, debate still persists about the relative importance of ARC versus POA kisspeptin neurons in triggering the onset of puberty in the female mouse.

### Rat

**Figure 2:**
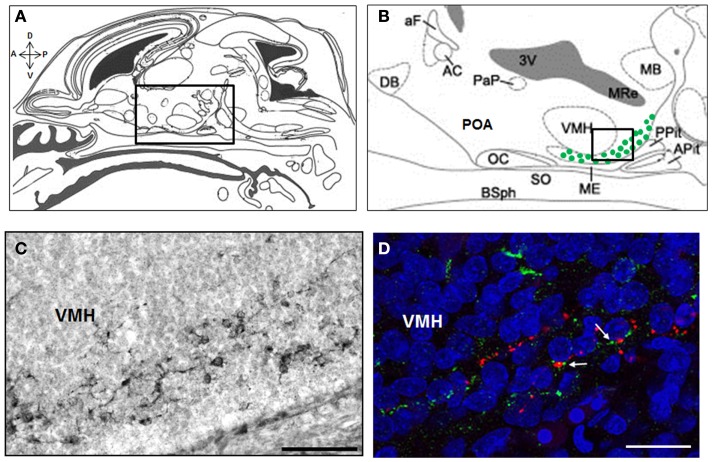
**Kisspeptin-immunoreactive cells and fibers in the ARC and median eminence at embryonic day E18.5 in rats**. Schematic mid sagittal view of embryonic head at E18.5 **(A)** (adapted from Paxinos and Ashwell, 2008) with a higher resolution of the embryonic hypothalamus **(B)** [corresponding to the box in **(A)**] depicting the area were kisspeptin-immunoreactive cells were detected (green dots). Numerous kisspeptin-immunoreactive cells were detected in the ARC at embryonic day (E) 18.5 as shown on this immunoperoxidase-labeled brain section with anti-kisspeptin AC067 **(C)** [corresponding to the box in **(B)**]. In the median eminence, close appositions (white arrows) between kisspeptin fibers (green) and GnRH fibers (red) were detected as shown in this confocal optical section of a slice double immunofluorescence-labeled for kisspeptin (sheep anti-5-18 kisspeptin 52 # AC067, Desroziers et al., 2012a) and GnRH (rabbit anti-2-10 GnRH # 19900, Caldani et al., 1988) and counterstained with Hoechst nuclear marker **(D)**. A, anterior; P, posterior; D, dorsal; V, ventral; DB, diagonal band of Broca; aF, anterior fornix; AC, anterior commissure; PaP, paraventricular nucleus; 3V, third ventricle; OC, optic chiasma; POA, preoptic area; SO, supraoptic nucleus; ME, median eminence; VMH, ventro-medial hypothalamus; MB, mammillary bodies; Pit, pituitary; Apit, anterior pituitary; Ppit, posterior pituitary; BSph, sphenoid bones; MRe, mammillary recess. Scale bars: 100 μm **(C)** and 20 μm **(D)**.

A recent study in the rat has provided strong foundation for our understanding of the embryonic stages of kisspeptin neuron development. Using a BrdU pulse chasing approach, we have established that the neurogenesis period of ARC kisspeptin cells in the female rat hypothalamus begins at about E12.5, peaks around E15.5, and is not yet over at E17.5 (Desroziers et al., 2012a). Using immunohistochemistry, we further show that some cells in the developing ARC already synthesize kisspeptins from about E14.5 (Desroziers et al., 2012a; Figure 2). The number of kisspeptin-immunoreactive cells in the fetal ARC increases between E14.5 and E18.5 which coincides with the time when GnRH fibers reach the portal vessels of the median eminence and circulating LH levels reach their maximum in the rat fetus (Ugrumov et al., 1985; Huhtaniemi, 1995). At E18.5, close appositions between kisspeptin- and GnRH-immunoreactive fibers can be detected in the median eminence (Figure 2D), consistent with the hypothesis that kisspeptins already control GnRH release prenatally. The number of kisspeptin-immunoreactive cells, as well as hypothalamic *Kiss1* mRNA levels, decrease at the end of gestation (Desroziers et al., 2012a). The mechanism of action and physiological relevance of this decrease have yet to be fully elucidated.

During postnatal development, *Kiss1* expression continues to be tightly regulated as shown in the pioneering study by Navarro et al. (2004b). In particular, real-time quantitative RT-PCR of *Kiss1* and *GPR54* content in the hypothalamus revealed a transient decline in the expression levels of both genes during the infantile period, followed by an increase around the time of puberty (Navarro et al., 2004b). These changes have since been spatially refined by studying the respective expression of these genes either by RT-PCR on tissue punches (Knox et al., 2009; Takase et al., 2009; Lederman et al., 2010; Li et al., 2012) or by *in situ* hybridization (Cao and Patisaul, 2011; Takumi et al., 2011; Patisaul et al., 2012). In the ARC, *Kiss1* expression is relatively high during the neonatal and early infantile period but decreases during the third postnatal week (Cao and Patisaul, 2011; Takumi et al., 2011). A decline in the density of kisspeptin-immunoreactive fibers has also been measured during that period of time (Desroziers et al., 2012b). These observations may be correlated to the reported transient activation of LH secretion during early postnatal life (Döhler and Wuttke, 1975). *Kiss1* expression in the ARC increases again at puberty (Knox et al., 2009; Takase et al., 2009; Takumi et al., 2011). This increase has been associated with an increase in basal circulating LH levels (Takase et al., 2009). Similarly, kisspeptin-immunoreactive cell numbers and fiber densities increase in the ARC during the peripubertal period (Desroziers et al., 2012b). As in mice, *Kiss 1* expression in the POA of rats is detected later than in the ARC, starting during the second postnatal week (Cao and Patisaul, 2011), and continues to increase until the fifth postnatal week (Desroziers et al., 2010; Takumi et al., 2011). By contrast to mice, however, no kisspeptin-immunoreactive cells has been detected so far in the POA throughout rat postnatal life, unless colchicine was administered (Takase et al., 2009; Desroziers et al., 2010, 2012b; Lederman et al., 2010). A very recent study on Sprague Dawley rats has enabled the peripubertal changes in *Kiss1* and *GPR54* expression in the ARC and POA to be assessed with greater time resolution and in relation to changes in the frequency of LH pulses (Li et al., 2012). No changes in *GPR54* expression are observed during this developmental time window but *Kiss1* mRNA levels significantly increase prior to puberty onset, first in the ARC and later in the POA. Notably, these changes are accompanied by an increase in LH pulse frequencies (Li et al., 2012). Our recent analysis of kisspeptin-immunoreactive fiber density across each of these two regions similarly suggest a sequential activation of kisspeptin-immunoreactivity at the time of puberty, first in the ARC, and second in the POA (Desroziers et al., 2012b). Collectively, these findings provide strong support for an important role of kisspeptins produced by ARC neurons in triggering puberty in female rats. During pubertal progression, this would be followed by an estrogen-dependent amplification system of GnRH release mediated by kisspeptins neurons of the POA.

In adult cycling females, the number of *Kiss1*-expressing cells varies across the estrous cycle, in opposite phases between the ARC and the POA (Smith et al., 2006a). Changes in *Kiss1* expression have also been evaluated across aging. In the rat, unlike in humans, reproductive senescence is associated with a decrease in the frequency and amplitude of GnRH/LH pulses and a progressive disappearance of GnRH/LH surges (Scarbrough and Wise, 1990). Consistently, a significantly lower number of kisspeptin-immunoreactive cells are detected in the POA of middle-aged rats compared to young rats, under estradiol positive feedback conditions (Lederman et al., 2010).

Thus, in the rat, *Kiss1* expression appears tightly regulated throughout the reproductive life cycle, not only in the POA, but also in the ARC where it is already detected prenatally. The developmental profile of *Kiss1* expression in the ARC and POA differ significantly. This may be related to different roles played by the two kisspeptin cell populations in the control of GnRH pulse amplitude and frequency.

### Sheep

Studies of the kisspeptin–GPR54 system in sheep have focused on two critical periods of development, the prenatal period and the peripubertal period. *Kiss1* expression has been detected by RT-PCR in the hypothalamus of 110 day old sheep fetuses (birth occurring around gestational day 145 in this species) both in rostral and caudal hypothalamic slices including the POA and ARC, respectively (Bellingham et al., 2009). This is a time when LH is already secreted in a GnRH-dependent, pulsatile manner (Matwijiw and Faiman, 1987). Thus, it is tempting to speculate that kisspeptins already provide tonic drive to GnRH neuronal activity *in utero*. *Kiss1* expression has further been studied by *in situ* hybridization in an estradiol-supplemented ovariectomized model of puberty (Redmond et al., 2011b). In this study, puberty was preceded by an elevation in the number of *Kiss1*-expressing neurons both in the POA and ARC. However, it is the increase in *Kiss1* expression levels in the ARC more specifically that could be correlated with an increase in LH pulse frequency (Redmond et al., 2011b). In intact ewes, the number of kisspeptin-immunoreactive cells in the ARC was shown to increase at puberty, concomitantly to an increase in LH pulse frequency but not pulse amplitude (Nestor et al., 2012). Over that same period of time, the proportion of GnRH neurons with close appositions of kisspeptin-immunoreactive fibers increase (Nestor et al., 2012), as previously reported in mice (Clarkson and Herbison, 2006). Whether this reflects a postnatal morphological plasticity of the system, or is related to enhanced *Kiss1* expression remains to be investigated. Taken together, these findings point to a potential relationship between postnatal changes in the kisspeptin–GPR54 system and the increase of LH secretion accompanying ovine puberty.

### Monkey

In the female monkey, hypothalamic *Kiss1* and *GPR54* expression levels have been monitored across puberty and aging. Real-time quantitative RT-PCR of *Kiss1* and *GPR54* mRNA levels in the mediobasal hypothalamus revealed that both of these transcripts significantly increase at puberty (Shahab et al., 2005). More recently, using an *in vivo* microdialysis method, the team of Ei Terasawa was able to show an increase in kisspeptin pulsatile release within the stalk median eminence during puberty (Guerriero et al., 2012b). During late puberty, *in vivo* secretion of kisspeptin is pulsatile and in almost perfect synchrony with GnRH pulses, a finding consistent with a possible role of kisspeptins in the tuning of GnRH pulsatility at puberty (Keen et al., 2008). Notably, no changes in the POA have yet been reported across monkey physiology. With aging, *Kiss1* and *GPR54* mRNA levels increase in the mediobasal hypothalamus but remain unchanged in the POA (Kim et al., 2009; Eghlidi et al., 2010). By analogy, LH pulsatile secretion increases in aging monkeys but these are still able to generate GnRH/LH surges (Gore et al., 2004). Collectively, these observations suggest that GnRH release in the monkey continues to be under the tight regulatory control of ARC and POA kisspeptins late in postnatal life.

### Human

A very recent study detected kisspeptin and GPR54 immunoreactivities in the hypothalamus of second trimester human fetuses (Guimiot et al., 2012). Kisspeptin-immunoreactivity declined at the end of gestation, as previously observed in rats (Desroziers et al., 2012a). A causal link between this decline and the decline in circulating levels of gonadotropins that was monitored in cord blood between the 30th week of prenatal life and birth was suggested (Guimiot et al., 2012). Postnatal analysis of *Kiss1* expression or kisspeptin-immunoreactivity in human brains has only been reported in post-pubertal subjects (Rometo et al., 2007; Hrabovszky et al., 2010, 2011). *Kiss1* expression in the infundibular nucleus has been shown to increase after menopause in women (Rometo et al., 2007). This increase may be related to the increase in GnRH/LH secretion characterizing human reproductive senescence (Kermath and Gore, 2012 for review). Again, these findings are consistent with the hypothesis that GnRH secretion in women continues to be regulated by kisspeptins late in life.

**Figure 3:**
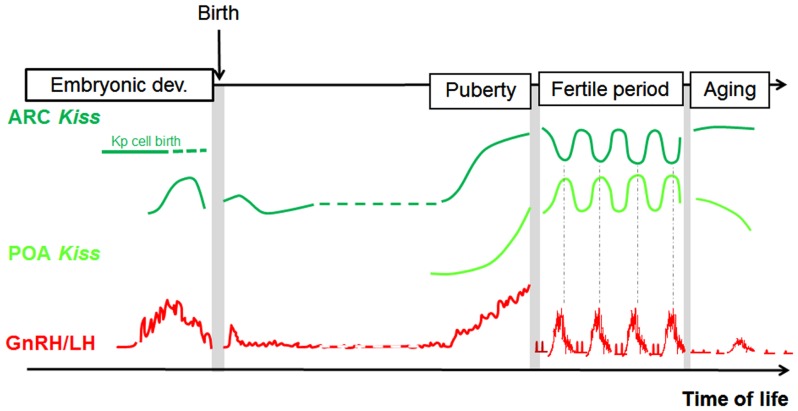
**Parallel developmental changes in *Kiss1* mRNA and circulating LH levels in rats**. Hypothetical scheme highlighting the parallel that can be made between Kiss1 expression in the POA and ARC and the profile of GnRH/LH release across different time windows of rat development (separated from each other by grey bars on the scheme because analyzed in separate studies). POA, preoptic area; ARC, arcuate nucleus; Kp, kisspeptin; LH, luteinizing hormone.

In conclusion, analogies can be found in each species between fluctuations in GnRH/LH levels and fluctuations in some aspects of the kisspeptin–GPR54 system, supporting the view that kisspeptin signaling in the brain controls GnRH secretion throughout life, including prenatally (Figure 3). The regulatory mechanism by which these changes in the kisspeptin–GPR54 system operate will be the focus of the following section.

## Endogenous Regulators of Kisspeptin–GPR54 during Development

### Developmental regulation of the kisspeptin–GPR54 system by gonadal steroids

Ever since the discovery that *Kiss1* expression is under tight regulation of estrogen receptor signaling (Smith et al., 2005) and that kisspeptin neurons express numerous sex steroid receptors in adult mice, rats, and sheep (Smith et al., 2005, 2006a, 2007; Franceschini et al., 2006; Adachi et al., 2007; Clarkson et al., 2008; Cheng et al., 2010), numerous studies have investigated the regulation of the kisspeptin–GPR54 system by gonadal steroids across development (Table 2).

**Table 2:** **Summary of studies showing an effect of sex steroids on the kisspeptin–GPR54 system throughout female development**.

Approaches	Species	Reference	Treatment	Period of treatment	Time of analysis	Hypothalamic impact	Physiological impact
Transgenetical reduction in sex steroid signalization	Mouse	González-Martínez et al. (2008)	Genetic deletion of alpha-fetoprotein (AFP-KO mice)	Late embryonic and neonatal period	Adult	Decrease in kp-ir cell numbers in the POA	Infertility
		Gill et al. (2010)	Mutation of GnRH receptor, resulting in reduced sex steroid signaling (Hpg mice)	Congenital	Peripubertal (P10, P30, P45) and adult	Decrease in Kiss1 mRNA and kp-ir cell numbers in the POA, increase of Kiss1 mRNA in the ARC	Absence of vaginal opening, infertility
		Mayer et al. (2010)	Targeted genetic deletion of estrogen receptor alpha in kisspeptin cells	Congenital	Peripubertal (P15, P25, P35) and adult	Decrease in kp-ir cell numbers in the POA, increase in Kiss1 mRNA in the ARC	Advanced vaginal opening		
		Clarkson et al. (2009), Bakker et al. (2010)	Genetic deletion of aromatase (ARKO: mice)	Congenital	Adult	Ovariectomy decreases Kiss1 mRNA and kp-ir cell numbers in the POA	Infertility
Gonadectomy ± sex steroid replacement	Mouse	Kauffman et al. (2009)	Ovariectomy	Prepubertal (PI4 and PI8) and adult	Prepubertal (P18) and adult	Decrease in Kiss1 cell numbers in the POA, increase in Kiss1 cell numbers in the ARC	Increase in circulating LH
		Clarkson et al. (2009)	Ovariectomy ± E2	Prepubertal (P15–P30 or P22–P30)	Prepubertal (P30)	Ovariectomy decreases kp-ir cell numbers in the POA. Effect reversed by E2 treatment	Increase in circulating LH. Circulating LH restored by E2
	Rat	Takase et al. (2009)	Ovariectomy ± E2	Prepubertal (P15–P21, P20–P26)	Peripubertal (P21, P26, P31, P36–41)	Ovariectomy suppresses the peripubertal increase in Kiss1 mRNA in the POA and ARC. Effect reversed by E2 treatment	Ovariectomy suppresses the peripubertal increase in circulating LH. Effect reversed by E2 treatment
		Takumi et al. (2012a)	Ovariectomy	Prepubertal (P14–P18)	Prepubertal (P18)	Decrease in Kiss1 cell numbers in the POA and ARC	
	Sheep	Nestor et al. (2012)	Ovariectomy	Pre- (20–24 W) and post-pubertal (>36 W)	Pre- (20–24 W) and post-pubertal (>36 W)	Increase in kp-ir cell numbers in the ARC, specifically at prepubertal stage	Increase in LH pulse frequency in prepubertal stage
	Monkey	Guerriero et al. (2012b)	Ovariectomy ± E2	Prepubertal (52–81 W) and pubertal (112–175 W)	Prepubertal (52–81 W) and pubertal (112–175 W)		Ovariectomy increases kp release specifically at pubertal stage. Effect reversed by E2 treatment
Pharmacological	Rat	Dickerson et al. (2011b)	EB injection	Embryonic (E16–E18)	Neonatal (P1) and adult	Decrease in kp-ir fiber density in the POA (adult)	Advanced vaginal opening, decrease in circulating LH
		Gore et al. (2011)	EB injection	Embryonic and neonatal (E19–P7)	Adult	Decrease in Kiss1 mRNA in the POA	Decrease in circulating E2, irregular estrus cycle
		Cao et al. (2012)	EB injection	Neonatal (P0–P2)	Neonatal (P4) and infantile (P10)	Decrease in Kiss1 mRNA from P4 in the ARC and from P10 in the POA	
		Navarro et al. (2004b), Navarro et al. (2009)	EB injection	Neonatal (P1)	Peripubertal (P30) and adult	Decrease in hypothalamic Kiss1 mRNA	Decrease in circulating LH
		Losa et al. (2011), Losa-Ward et al. (2012), Patisaul et al. (2012)	EB or E2 injection	Neonatal (P0–P3)	Peripubertal (from P17 to P33)	Decrease in Kiss1 mRNA and kp-ir in the POA and ARC	Advanced vaginal opening
		Kauffman et al. (2007b)	TP injection	Neonatal (P1)	Adult	Decrease in Kiss1 cell numbers in the POA but not in the ARC	Decrease in circulating LH
		Bateman and Patisaul (2008), Patisaul et al. (2009), Patisaul et al. (2012)	EB injection	Neonatal (P0–P3)	Adult	Decrease in kp-ir in the POA and ARC	Irregular estrus cycles, decrease in circulating LH
	Sheep	Cheng et al. (2010)	TP injection	End of gestation	Adult	No change in kp-ir cell numbers in the POA and ARC	Increase in circulating LH and LH pulse frequency

*POA, preoptic area; ARC, arcuate nucleus; GnRH, gonadotropin-releasing hormone; LH, luteinizing hormone; kp-ir, kisspeptin-immunoreactive; EB, estradiol benzoate; TP, testosterone propionate; E2, estradiol; E, embryonic day; P, postnatal day; W, weeks*.

#### Mouse

Female mice display a much greater number of kisspeptin-immunoreactive cells in the POA than male mice (Clarkson and Herbison, 2006) even after gonadectomy (González-Martínez et al., 2008), suggestive of organizational effects of sex steroids on this cell population during development. This sexual dimorphism appears to develop between postnatal days 10 and 12, that is shortly after the first *Kiss1*-expressing cells can be detected in this region (Semaan et al., 2010). It does not appear to involve bax-mediated apoptosis (Semaan et al., 2010) but differential transcription patterns of the *Kiss1* gene governed by sex-specific epigenetic mechanisms during development (Semaan et al., 2012). For instance, the methylation pattern of the *Kiss1* gene in the POA (but not the ARC) significantly differs at several CpG sites between males and females (Semaan et al., 2012). The contribution of estrogen signaling to the sexual differentiation of the kisspeptin cell population of the POA has mostly been explored by the means of genetic manipulations. Mice genetically deficient in alpha-fetoprotein (AFP-knock-out) have excess estradiol signaling in the brain during the perinatal period. Female AFP-knock-out mice develop as few kisspeptin cells in the POA as males and their circulating LH levels are not increased by estradiol and progesterone-induced positive feedback conditions (González-Martínez et al., 2008). Thus, it may be that estradiol signaling in the brain at the time of the perinatal testosterone surge may be in part responsible for the defeminization/masculinization of the POA kisspeptin cell population in males. Interestingly, transgenic reduction of developmental sex steroid hormone signaling in a variety of mouse models also results in a marked decrease in the adult number of kisspeptin-immunoreactive neurons in the POA (Clarkson et al., 2009; Bakker et al., 2010; Gill et al., 2010; Mayer et al., 2010). For example, in the adult hypogonadal hpg mice, the number of kisspeptin-immunoreactive cells in the POA is reduced by half and it is not possible to increase this number by estradiol administration as it is for wild type mice (Gill et al., 2010). In aromatase knock-out (ArKO) mice which completely lack the capacity to synthesize estrogens, the number of kisspeptin-immunoreactive cells in the POA is also significantly reduced compared to wild type mice (Clarkson et al., 2009), even under identical positive feedback conditions (Bakker et al., 2010). In the same vein, ArKO mice, whether ovariectomized or treated with estradiol and/or progesterone to mimic positive feedback conditions, display fewer *Kiss1*-expressing cells in the POA compared to wild type mice (Szymanski and Bakker, 2012), consistent with the hypothesis that postnatal estrogens may reprogram *Kiss1* transcription in the POA. Furthermore, while odors from male urine are able to induce c-fos expression in a good proportion of POA kisspeptin cells in wild type female mice, this proportion is markedly reduced in the ArKO mice (Bakker et al., 2010). Data from experimental gonadectomy and estradiol replacement during development suggest that estrogens stimulate kisspeptin expression in the POA before puberty (Clarkson et al., 2009). These authors proposed that this estrogen-dependent increase of kisspeptin expression in the POA may facilitate the emergence of pulsatile gonadotropin secretion necessary for puberty onset, as well as the preovulatory estrogen-dependent surge (Clarkson et al., 2009). However, it was recently shown that ArKO mice maintain the ability to mount an LH surge when treated with hormones mimicking positive feedback conditions in adulthood, questioning the absolute requirement of the full female-typical (estrogen induced) complement of POA kisspeptin neurons in generating GnRH/LH surge (Szymanski and Bakker, 2012).

In the mouse ARC, the sexual dimorphism of the kisspeptin cell population is not as obvious as in the POA. In this nucleus, female mice display a greater number of *Kiss1*-expressing cells than males on the day of birth but this difference is no longer visible during infancy or in adulthood (Kauffman et al., 2009; Poling and Kauffman, 2012). By gonadectomizing the mice at different stages of development, Kauffman et al. (2009) revealed an important gonadal hormone-independent sex difference in the number of ARC *Kiss1* cells during the infantile period. Prepubertal gonadal hormones appear to exert a greater repressive action on ARC *Kiss*1 expression in females than in males (Kauffman et al., 2009). Of interest, this strong downregulation of *Kiss1* expression in the prepubertal female ARC most likely occurs through Erα signaling within kisspeptin cells themselves and may be of physiological relevance in the timing of puberty (Mayer et al., 2010). Indeed, female mice with a targeted deletion of *Esr1* within kisspeptin cells not only display enhanced *Kiss1* expression in the ARC at the juvenile stage but also a dramatic advance in the timing of vaginal opening. Moreover, circulating LH levels in these genetically modified mice are higher than in control mice (Mayer et al., 2010). These authors proposed that the prepubertal repressive action of estrogen signaling on *Kiss1* expression in the ARC represents an essential break to the central activation of the gonadotropic axis, preventing premature puberty onset (Mayer et al., 2010).

Collectively, these findings suggest that in the mouse, estrogen signaling may exert organizational effects on the kisspeptin cell population of the POA during the perinatal period in males and during the prepubertal period in females. Further studies are needed to elucidate the developmental origin of the sexual dimorphism observed at the level of ARC kisspeptin cell population. In particular, it is still unclear whether the sex-specific pattern of *Kiss1* expression detected in the absence of gonadal hormones during infancy in this nucleus is conditioned by a sex-specific and gonad-independent developmental program or by an organizational effect of the perinatal testosterone surge.

#### Rat

The number of POA *Kiss1*-expressing cells is greater in female rats than in male rats under identical estrogen positive feedback conditions, suggesting that, like in mice, developmental sex steroids may have important organizational effects on this cell population (Kauffman et al., 2007b; Homma et al., 2009). The importance of the perinatal period in this process is suggested from several studies using gain or loss of function approaches. Male pups neonatally orchidectomized develop a female-specific pattern of *Kiss1* expression in the POA (Homma et al., 2009; Takumi et al., 2012a). Conversely, female pups exposed to synthetic estrogens or aromatizable androgens such as estradiol benzoate or testosterone propionate during the perinatal period display a male-specific pattern of *Kiss1* expression or kisspeptin-immunoreactivity in adulthood (Navarro et al., 2004b; Kauffman et al., 2007b; Homma et al., 2009; Dickerson et al., 2011a).

By contrast to the POA, similar numbers of *Kiss1*-expressing cells and similar levels of *Kiss1* transcripts have been detected in the ARC of gonadectomized male and female rats (Adachi et al., 2007; Kauffman et al., 2007b; Homma et al., 2009). Furthermore, the downregulating effect of estradiol on *Kiss1* transcription in this nucleus appears similar in both sexes (Adachi et al., 2007; Kauffman et al., 2007b; Homma et al., 2009). In the ARC of intact adult rats, however, several studies have shown that females display more *Kiss1*-expressing cells or kisspeptin-immunoreactivity than males (Iijima et al., 2011; Takumi et al., 2011; Desroziers et al., 2012b). A real-time quantitative RT-PCR analysis of *Kiss1* transcript content in the ARC also points to the fact that the sex difference in *Kiss1* transcript levels is more or less visible, depending on the stage of the estrus cycle (Adachi et al., 2007). This sex difference develops during the neonatal period (Cao and Patisaul, 2011; Desroziers et al., 2012b) and its amplitude appears to fluctuate across postnatal development (Cao and Patisaul, 2011; Takumi et al., 2011; Desroziers et al., 2012b), presumably reflecting different developmental fluctuations in circulating gonadal hormones between males and females, as well as sex-specific developmental changes in the sensitivity of ARC kisspeptin cells to these hormones. For instance, data from experimental ovariectomy and estradiol replacement across different developmental time windows suggest that the regulatory action of estrogen signaling on *Kiss1* expression may change at the time of female puberty, leading to a developmental increase of *Kiss1* mRNA levels in both regions, and to an increase in circulating LH levels (Takase et al., 2009). Accordingly, we recently measured a peripubertal increase in kisspeptin-immunoreactivity within both regions at a time when peripheral estradiol levels were relatively constant (Desroziers et al., 2012b).

Another period of life when the sensitivity of kisspeptin cells to the feedback action of ovarian hormones appears to change is during aging. Middle-aged female rats that are gonadectomized and supplemented with estradiol benzoate and progesterone to mimic positive feedback conditions display an attenuated rise in the number of kisspeptin-immunoreactive cells in the POA as compared to young females under the same conditions (Lederman et al., 2010). By contrast, the repressive action of these gonadal hormones on ARC *Kiss1* mRNA and kisspeptin levels appears unaffected by aging (Lederman et al., 2010).

Collectively, these results suggest that estrogen signaling during the neonatal period organizes sex differences in *Kiss1* expression in the POA and that the sensitivity of both kisspeptin cell populations to estrogen signaling is dynamic across the lifespan of female rats.

#### Sheep

Female sheep display a greater number of kisspeptin-immunoreactive cells than male sheep not only in the POA but also in the ARC (Cheng et al., 2010). This sex difference in the ARC is also observed in pubertal sheep after gonadectomy, suggesting that it may result from organizational effects of gonadal steroids (Nestor et al., 2012). In this precocious species, sexual differentiation of the neuroendocrine circuits controlling reproductive function is mainly under the organizational influence of prenatal androgens during fetal life. Accordingly, female sheep treated prenatally with androgens display a lower responsiveness of the GnRH system to the negative feedback influence of progesterone. This translates into increased LH pulse frequencies and mean concentrations in peripheral blood (Cheng et al., 2010). However, this prenatal androgenization does not alter the number of kisspeptin-immunoreactive cells in the ARC nor in the POA. Rather, it is the number of cells immunoreactive for dynorphin or NKB, two other neuropeptides co-expressed with kisspeptins in the ARC, that is reduced compared to control females (Cheng et al., 2010). It may be that a greater exposure to androgens is required (dose and length) or that the influence of sex-specific genetic factors prevail in the sexual differentiation of the ovine kisspeptin system. Around the time of puberty, the negative feedback action of gonadal hormones on ARC kisspeptin-immunoreactivity diminishes parallel to an increase in LH pulse frequencies, suggesting, as in the rat, that a change in the sensitivity of ARC kisspeptin cells to the negative feedback action of estradiol signaling may initiate puberty onset (Nestor et al., 2012).

#### Monkey

There is to our knowledge no published information of a clear sexual dimorphism of the kisspeptin system in the monkey. In the female monkey, the positive and negative feedback action of estradiol on the kisspeptin–GPR54 system have been studied at the time of puberty (Guerriero et al., 2012b) and during aging (Eghlidi et al., 2010). Prepubertal ovariectomy does not prevent the peripubertal rise in kisspeptin pulsatile release within the median eminence. In fact, the repressive action of estradiol on this release appears to develop later, once puberty has already started (Guerriero et al., 2012b). Ovariectomy and short-term estradiol replacement appears to have little effect on ARC *Kiss1* expression levels in young monkeys (Eghlidi et al., 2010). By contrast, long-term ovariectomy (mimicking the loss of ovarian steroid negative feedback that occurs during perimenopause) significantly increases ARC *Kiss1* expression levels. *Kiss1* expression levels can be restored to normality by short-term estradiol supplementation (Eghlidi et al., 2010). Collectively, these results minimize the role played by estradiol signaling in the upregulation of kisspeptin release at puberty in the female monkey but suggest a role of this signaling in the upregulation of *Kiss1* expression in the ARC with aging.

#### Human

Little is known in humans about the impact of sex steroids on the developing kisspeptin–GPR54 system. In adult women, the number of kisspeptin-immunoreactive neurons is greater than in men both in the infundibular nucleus and in the POA (Hrabovszky et al., 2010). However, the involvement of developmental sex steroids in this dimorphism is yet to be defined.

### Developmental regulation of kisspeptin neurons by other factors

**Figure 4:**
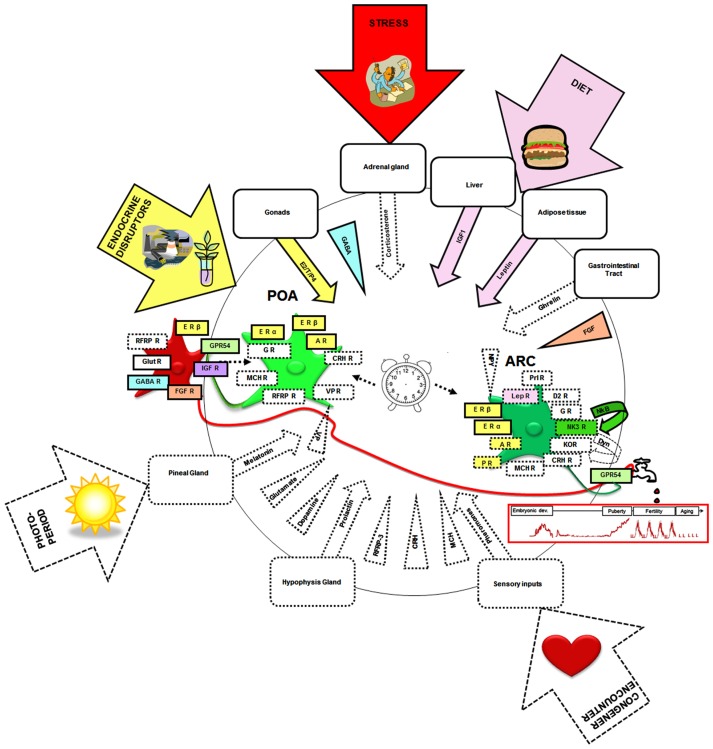
**Neural, hormonal, and environmental factors regulating the kisspeptin–GPR54 system**. Scheme summarizing the different factors that have been shown to regulate the kisspeptin–GPR54 system only during adulthood (squared by dashed lines) or also during development (squared by full lines and colored). Hormonal factors are codified by an arrow and central factors by a triangle. Molecular factors have been included whose receptors have been found on some kisspeptin neurons, factors found within fibers in close apposition to kisspeptin neurons, factors eliciting c-fos expression, or an electrophysiological response within kisspeptin neurons or changing *Kiss1* or *GPR54* mRNA levels, kisspeptin or GPR54 immunoreactivities, or the number of Kiss1/kisspeptin expressing cells when exogenously administered. Of note this synthetic scheme combines data from mice, rats, sheep, and monkeys and therefore occults potential species differences that may exist in these regulations. It is hypothesized that the developmental pattern of GnRH release (red graph below the tap) is shaped by interactions of these different neural and hormonal factors with an intrinsic differentiation program of the system (central clock). The developing kisspeptin–GPR54 system is particularly vulnerable to some environmental factors like endocrine disruptors, diet, and stress which can alter GnRH secretion and reproductive function on the long-term. POA, preoptic area; ARC, arcuate nucleus; E2, estradiol; T, testosterone; P4, progesterone; ER, estrogen receptor; AR, androgen receptor; PR, progestin receptor; IGF, insulin-like growth factor; IGF-R, insulin-like growth factor receptor; FGF, fibroblast growth factor; FGF-R, fibroblast growth factor receptor; GABA-R, GABA receptor; RFRP3, RF-amides related peptide-3; RFRP3-R, RFRP3 receptor; LepR, leptin receptor; Prl-R, Prolactin receptor; NKB, neurokinin B; NK3R, NKB receptor; Glut-R, glutamate receptor; VP, vasopressin; VP-R, vasopressin receptor; MCH, melanocortin; MCH-R, MCH receptor; Dyn, dynorphin; KOR, kappa-opioid receptor (Dyn-receptor); GR, Glucocorticoid receptor; CRH, corticotrophin-releasing hormone; CRH R, corticotrophin-releasing hormone receptor; D2-R, dopamine-receptor. The illustrations in the arrows were obtained from Clipart Microsoft Word^®^.

In the adult, there exists neuroanatomical, electrophysiological, and/or pharmacological evidence that the kisspeptin–GPR54 system may be the target of a variety of other hormones, neurotransmitters, and neuropeptides (Figure 4, squared by dotted lines). These include leptin (Smith et al., 2006b; Backholer et al., 2010), corticosterone (Kinsey-Jones et al., 2009), prolactin (Li et al., 2011), melanocortin hormone (Cravo et al., 2011), pheromones (Bakker et al., 2010), melatonin (Revel et al., 2006), ghrelin (Forbes et al., 2009), melanin-concentrating hormone (Wu et al., 2009; Cravo et al., 2011), corticotropin-releasing hormone (Takumi et al., 2012b), vasopressin (Vida et al., 2010), glutamate (Ducret et al., 2010), GABA (García-Galiano et al., 2012a), dopamine (Goodman et al., 2012), NKB (Navarro et al., 2011), and RFRP3 (Rizwan et al., 2012). However, only a few of these molecular factors have yet been identified as potential actors in the developmental regulation of the kisspeptin–GPR54 system (Figure 4, squared by full lines).

#### Leptin

Among hormonal factors, leptin, a well known permissive factor for pubertal maturation, is a potential upstream regulator of the kisspeptin–GPR54 system. Leptin deficient ob/ob mice display a marked reduction of *Kiss1* expression in the ARC and in the number of kisspeptin-immunoreactive cells in the POA but it is not yet known when exactly during development this effect starts (Quennell et al., 2011). The gonadotropin response of prepubertal rats to acute central administration of kisspeptin is preserved in different models of leptin deficiencies such as after central immunoneutralization of leptin or food restriction and in leptin resistant Zucker rats, indicating that leptin must act upstream of kisspeptin signaling (Navarro et al., 2004a; Castellano et al., 2011). In prepubertal rats under different food regimens, a positive correlation has been found between circulating leptin levels and hypothalamic *Kiss1* and *GPR54* mRNA levels (Iwasa et al., 2010a; Castellano et al., 2011). Pharmacological manipulations of mTOR, a transducer of leptin’s effect on energy homeostasis, suggested that this signaling pathway is essential for activating *Kiss1* transcription in the ARC at puberty onset (Roa et al., 2009). However, administration of leptin was able to restore hypothalamic levels of *GPR54* but not *Kiss1* mRNA in prepubertal rats displaying reduced levels of both *GPR54* and *Kiss1* mRNA after 24 h food deprivation (Iwasa et al., 2010a). The precise site of leptin’s action in the brain during the pubertal transition period may vary between species and remains a matter of debate (Elias and Purohit, 2013).

#### Neuropeptides

Among neuropeptides potentially regulating the kisspeptin–GPR54 system during development, a particular attention has been paid to NKB. Indeed, human genetic studies have associated loss of function mutations on either TAC3 or TACR3 genes (encoding NKB and its receptor NK3R respectively) with hypogonadism and infertility, similar to the phenotypes of *Kiss1* or *GPR54* mutants (Topaloglu et al., 2009). Interestingly, NKB receptors have been found on kisspeptin neurons of the ARC, which also co-express NKB, but not on GnRH neurons (Navarro et al., 2012). In prepubertal hpg mice, administration of a specific NK3R antagonist showed that NKB does not control *Kiss1* expression (Gill et al., 2012). Instead, there is increasing evidence that this neuropeptide stimulates kisspeptin release. For instance, the LH releasing activity of the NKB agonist senktide is abolished in *GPR54* knock-out mice (García-Galiano et al., 2012b), in the presence of a GPR54 antagonist in prepubertal rats (Grachev et al., 2012) or after GPR54 desensitizing in agonadal juvenile monkeys (Ramaswamy et al., 2011). A model has recently been proposed where NKB would participate in the initiation of pulses of kisspeptin release by synchronizing ARC kisspeptin neuronal activity through an autofeedback loop (Navarro, 2012).

Another neuropeptide that may cross-talk with the kisspeptin–GPR54 system during development is RFRP3. This mammalian ortholog of GnIH is already found closely apposed to a large proportion of GnRH neurons in prepubertal rats (Losa-Ward et al., 2012) where it may act by decreasing the GnRH neuronal response to kisspeptins as previously shown by electrophysiological recordings on brain slices from adult mice (Wu et al., 2009). In addition, RFRP3 may act upstream of kisspeptin neurons in light of a recent study in hamster where this peptide has been proposed to convey a melatoninergic signal to GnRH neurons through regulation of *Kiss1* transcription (Ancel et al., 2012).

#### Growth factors

The developing kisspeptin–GPR54 system may also be the target of growth factors: mice harboring deficiencies in FGF8 and/or FGFR-1 display a greater number of kisspeptin-immunoreactive cells in the POA at some stages of peripubertal development specifically, suggesting that FGF signaling pathways may control kisspeptin cell numbers or act on the *Kiss1* gene at transcriptional or post-transcriptional levels (Tata et al., 2012). Another growth factor that may be implicated during development of kisspeptin neurons is IGF1. Female rats that receive an intracerebroventricular administration of IGF1 during the prepubertal period display increased *Kiss1* mRNA levels in the POA specifically. This effect can be abolished by the administration of an IGF1 receptor antagonist and appears dependent on the presence of gonadal estrogens (Hiney et al., 2009). This contrasts with results obtained in adulthood where the same antagonist produces no effect on *Kiss1* expression, suggesting that the IGF1 receptor response of kisspeptin neurons may change as development proceeds (Todd et al., 2007).

#### GABA

In the female rhesus monkey, a very recent study demonstrated the fundamental role of GABA signaling in restraining kisspeptin release prior to puberty. GABA_A_ receptor antagonist administration during the prepubertal period but not during the pubertal period stimulates kisspeptin release in the medial basal hypothalamus (Kurian et al., 2012). In the same study, the use of a GPR54 antagonist suggested that kisspeptin neurons may relay inhibitory GABA signals to GnRH neurons prior to puberty. In addition, the response of GnRH neurons to kisspeptins can be modulated by GABA signaling in adult mice and rats and it will be interesting to further explore when during development this cross-talk is established (Pielecka-Fortuna and Moenter, 2010; García-Galiano et al., 2012a).

#### Transcription factors

The concept has recently been put forward that puberty is controlled by regulatory gene networks composed of multiple functional modules operating with overlaps of partially redundant pathways (Ojeda et al., 2010). In this context, there has been a great interest in positioning the *Kiss1* gene within a framework of puberty-associated identified transcription factors. *In vitro* promoter assays in human cell lines suggest that the *Kiss1* gene is regulated by a set trans-activators and repressors involved in the system-wide control of mammalian puberty, among which TTF1, CUX1-p200, EAP1, YY1, and CUX1-p110 (Mueller et al., 2011). It will be of great interest to confirm the relevance of these findings *in vivo*.

## The Developing Kisspeptin–GPR54 System as Target of Environmental Disruptors of Reproduction

### Endocrine disruptors

A variety of endocrine disrupting chemicals (EDCs) have recently been shown to disrupt the orderly progression of the female reproductive life cycle in association with changes in the development of the kisspeptin–GPR54 system. Most studies reporting alterations of the kisspeptin–GPR54 system by EDCs have been performed in rats. For example, estradiol benzoate, genistein, and polychlorinated bisphenyls (PCBs), if administered during rat perinatal development, have each been shown to advance vaginal openings and accelerate reproductive senescence, associated with a reduction in hypothalamic kisspeptin-immunoreactivity in adulthood (both at the level of the POA and ARC). This reduction in kisspeptin-immunoreactivity is observed after normalization of sex steroid circulating levels and is associated with a decline in the proportion of GnRH neurons being activatable by hormonal stimuli mimicking estradiol positive feedback conditions (Bateman and Patisaul, 2008; Dickerson et al., 2011a; Patisaul et al., 2012). Long Evans rats administered with estradiol benzoate or genistein during the first 4 days of life have further been analyzed for kisspeptin-immunoreactivity around puberty (Losa et al., 2011). A significant reduction in kisspeptin-immunoreactivity (both at the level of the POA and ARC) was detected in these EDC-treated animals compared to control rats. A recent *in situ* hybridization analysis shows that female Long Evans rats neonatally administered with estradiol benzoate display lower *Kiss1* hybridization signals than controls in both brain regions during the pubertal transition period (Patisaul et al., 2012). This reduction has been evidenced as early as postnatal day 4 in the ARC and postnatal day 10 in the POA (Cao et al., 2012), suggesting that the *Kiss1* gene may represent an early target of this endocrine disruptor in the hypothalamus. By postnatal day 10, sex differences in *Kiss1* mRNA signal in the ARC are no longer observed following neonatal administration of estradiol benzoate (Cao et al., 2012). Interestingly, this study also suggests that a neonatal exposure to estradiol benzoate can induce a rapid downregulation of *Esr1* and *Esr2* transcript levels in various hypothalamic nuclei, including the POA and ARC (Cao et al., 2012). Of note, other hypothalamic targets of estradiol benzoate have recently been evidenced in the POA of newborn Sprague Dawley rats that had been exposed *in utero* to this endocrine disruptor, using a 48 gene TaqMan PCR-based array (Dickerson et al., 2011b). For instance, on the first day of postnatal life, the gene encoding prodynorphin was shown to be upregulated and genes encoding subtypes of NMDA and GABA receptors downregulated in the POA (Dickerson et al., 2011b). A similar result was obtained following prenatal PCB exposure (Dickerson et al., 2011b). At the age of 2 months, 4 POA genes out of 48 appear significantly reduced to male levels in the EDC-exposed females, including the androgen receptor, NMDA receptor 2b, IGF1, and TGFβ1. It is noteworthy that all four of these genes play roles in hypothalamic development, including in the regulation of GnRH and kisspeptin neurons (Hiney et al., 2009; Oakley et al., 2009; Kurian et al., 2012). However, no changes in *Kiss1* expression levels could be detected in this study, despite the reduction in kisspeptin-immunoreactivity observed (Dickerson et al., 2011a). This may be due to a methodological limitation or to a different regulation between the mRNA and the protein. The same approach has been used to examine the molecular consequences in the POA of the aged female progeny, of a perinatal exposure to estradiol benzoate, a treatment that results in an acceleration of reproductive aging in Fisher rats (Gore et al., 2011). A complex regulatory neural/glial network of 17 genes controlling reproductive function appears upregulated in the POA by this perinatal estradiol benzoate treatment, including sex steroid hormone receptors, GABA and glutamate receptor subunits, growth factors, neuropeptide receptors, and a transcription factor. The *Kiss1* gene appears significantly downregulated and *Esr1* upregulated, relative to controls. Interestingly, an increase in methylation at some CpG sites in the *Esr1*gene is observed, suggesting that early exposure to this endocrine disruptor can induce lifelong epigenetic changes in this gene (Gore et al., 2011). It will be of great interest to further assess whether endocrine disruptors can also interfere with epigenetic marks on the *Kiss1* gene (Semaan et al., 2012) and to position *Kiss1* within this identified network of EDC-sensitive hypothalamic genes.

Bisphenol A (BPA), another endocrine disruptor with both estrogenic and anti-androgenic activities, may also target the kisspeptin–GPR54 system during development. However, studies are sparser and often incomplete, making it difficult to draw a clear picture of its mechanism of action. Administration of high (but not low) doses of BPA to neonatal female Long Evans rat results in adulthood in decreased kisspeptin-immunoreactivity in the ARC, independently of the steroidogenic milieu (Patisaul et al., 2009). If administered at high doses to neonatal Wistar rats, BPA induces a decrease in *Kiss1* hypothalamic expression levels, as well as a decrease in kisspeptin-immunoreactivity in the ARC at puberty (Navarro et al., 2009; Losa-Ward et al., 2012). If administered at low doses to neonatal Wistar rats, it advances the time of vaginal opening without any changes in kisspeptin-immunoreactivity being detectable in the ARC or POA at puberty. Finally, a low dose neonatal administration to Long Evans rats has recently been shown to decrease *Kiss1* hybridization signals in the POA as early as P10 (Cao et al., 2012). In CD1 mice, a lifelong-exposure to high doses of BPA results in an increase rather than a decrease in hypothalamic *Kiss1* mRNA levels (Xi et al., 2011). Furthermore, BPA-exposed mice display higher levels of circulating estradiol than control mice (Xi et al., 2011). Similarly, a perinatal exposure of female CD1 mice to extremely low levels of BPA results in an increase in the number of kisspeptin-immunoreactive cells in the POA (Panzica et al., 2011). These opposite effects of BPA between mice and rats illustrate the species-specificity of the effect of endocrine disruptors in general.

The consequences of real life exposure to EDCs was furthermore assessed in a farm animal species, the sheep. The fetuses (110 day old) of pregnant ewes exposed from the first day of conception to sewage sludge containing common endocrine disruptors display lower *Kiss1* mRNA levels in the ARC and in the POA relative to control animals maintained on pasture treated with conventional inorganic fertilizers (Bellingham et al., 2009). The physiological and behavioral outcomes of this exposure remains to be assessed.

Whether there is a causal link between disruption of the kisspeptin–GPR54 system and the different reproductive defects engendered by these EDCs remains to be fully investigated. Many recent studies on neuroendocrine disruption of reproductive function have focused on analysis of the kisspeptin–GPR54 system as potential target but it is clear that developmental exposure to some endocrine disruptors can advance the time of vaginal opening independently of kisspeptin signaling (Witham et al., 2012). This may in some cases derive from direct peripheral effects of the EDC or through interferences with other steroid-sensitive neural circuits regulating GnRH secretion. For example, neonatal exposure of female rats to low levels of BPA was recently found to advance the time of vaginal opening and has been associated to a decrease in the number of RFRP3-immunoreactive neurons and in the proportion of GnRH neurons displaying RFRP3-immunoreactive fiber appositions (Losa-Ward et al., 2012). On the other hand, no changes in kisspeptin-immunoreactivity could be detected (Losa-Ward et al., 2012).

### Diet

It has been known for a long time that diet can have a strong impact on reproductive function, including on the timing of puberty onset. This observation has led several laboratories to investigate a potential regulatory role of diet on the development of the kisspeptin–GPR54 system. In mice, a high fat diet given from the time of weaning can induce infertility in the DJA strain but not in the C57/Bl6 strain. Accordingly, this food regimen results in adulthood in a decrease of ARC and POA *Kiss1* expression specifically in the DJA strain (Quennell et al., 2011). Mouse nutrition has also been manipulated during early postnatal development by varying litter sizes during lactation from postnatal day 4 until weaning and this was shown to produce long-lasting changes in kisspeptin-immunoreactivity and in physiological parameters (Caron et al., 2012). Under-nourished pups raised in large litters during lactation display delayed puberty onset and reduced fertility index and this has been associated with a decrease in the density of fibers double labeled for kisspeptin and NKB in medial preoptic nuclei (Caron et al., 2012). DiI anterograde tracing studies suggest that this results from a neonatal impairment of kisspeptin neural projections from the ARC toward the medial preoptic nucleus (Caron et al., 2012).

In rats, chronic undernutrition from the time of weaning onward (Castellano et al., 2005; Navarro et al., 2012) diminishes hypothalamic *Kiss1* mRNA levels around puberty. This is associated with delayed onset of vaginal opening, and decreased circulating levels of LH, effects that can be rescued by prepubertal chronic daily central administration of either kisspeptin (Castellano et al., 2005) or senktide, a NKB agonist (Navarro et al., 2012). Chronic undernutrition exclusively during the fetal period (Iwasa et al., 2010b) or during lactation (Castellano et al., 2011) is sufficient to induce long-term changes, at least until the pubertal period: puberty onset is again delayed and hypothalamic *Kiss1* mRNA levels are decreased. Neuroanatomical analysis further showed a decrease in the number of kisspeptin immunoreactive cells in the ARC following undernutrition during lactation (Castellano et al., 2011). Conversely, overnutrition by litter size manipulation during lactation increases hypothalamic *Kiss1* mRNA levels at the pubertal transition period, advances puberty onset, and increases kisspeptin-immunoreactive fiber density in the POA (Castellano et al., 2011). High fat diet from the time of weaning can also advance puberty onset and results in an increase in *Kiss1* mRNA levels in the ARC prior to vaginal opening, followed by a decrease in the POA (Li et al., 2012).

### Stress

The functioning of the hypothalamic–pituitary–gonadal axis can also be altered by stressful experiences early in life. Female rats that are exposed to an immunological stress before 7 days of postnatal life exhibit a significant delay in puberty-associated with decreased *Kiss1* but not *GPR54* expression in the POA (Knox et al., 2009). Thus, kisspeptin neurons may represent important cellular relays through which stress-related factors impact the reactivation of GnRH pulsatile release at puberty. Notably, corticotropin-releasing factor receptors and glucocorticoid receptors have recently been detected by immunohistochemistry in kisspeptin neurons of the ARC in adulthood (Takumi et al., 2012b), implying a possible direct effect of stress-related factors on kisspeptin neurons. It will be interesting to determine when during development expression of these receptors start.

## Conclusion

Loss and gain of function studies during development have now provided compelling evidence that kisspeptin signaling in the brain is essential for the maturation of reproductive function through puberty in several mammalian species including humans. Upregulation of *Kiss1* transcription both in the ARC and POA appears to play a major role in the onset and progression through puberty in several mammalian species. Numerous potential regulators of *Kiss1* transcription during development have been identified and light has also recently been shed on developmental regulators of kisspeptin release, including NKB and GABA. Nevertheless, a lively debate still persists on the respective roles played by the ARC and POA populations of kisspeptin neurons in puberty onset, progression, and completion. For example, *Kiss1* expression or kisspeptin-immunoreactivity does not appear to increase in the ARC at the time of puberty in mice, as opposed to rats, sheep, and monkeys for which positive correlations have been found between *Kiss1* mRNA levels in the ARC and LH pulse frequencies. On the other hand, the mouse is the only species for which an upregulation of *GPR54* expression has been described in GnRH neurons during female postnatal development. Since studies using different species often rely on different approaches with different sensitivities, it may be hazardous at this stage to put these different observations at the account of true species differences. Clearly, further investigations using a variety of complementary approaches on each animal model are needed in order to identify and ascertain species-specific processes in the developmental regulation and function of the kisspeptin–GPR54 system.

Numerous studies have shown that the kisspeptin–GPR54 system is particularly sensitive to gonadal steroids. In fact, all species studied develop a clear sexual dimorphism in the pattern of *Kiss1* expression in the POA (with greater expression in females) but the precise roles of developmental sex steroids in this process have so far only been studied in mice and rats using different yet complementary approaches. Estrogen receptor signaling appears to exert important organizational effects during the perinatal and peripubertal periods that may involve epigenetic regulations of the *Kiss1* and/or *Esr1* genes. In mice, rats, sheep, and humans, sex differences in the amounts of *Kiss1* expression and/or kisspeptin-immunoreactivity have also been detected in the ARC at some points of development (different depending on species) but the respective roles played by organizational and activational effects of sex steroids in these sex differences remain to be fully analyzed and documented. In mice, rats, and sheep, different yet dynamic sensitivities of male and female ARC kisspeptin cells to circulating sex steroids have been highlighted at the time of puberty. It seems clear that estrogen signaling can exert both organizational and activational effects on the kisspeptin–GPR54 system albeit at times of development and at cellular levels that can greatly vary between species and strains. More detailed studies on the mechanism of action of sex steroids on the development of the kisspeptin–GPR54 system with its neuroendocrine and behavioral consequences should be conducted in the future.

Moreover, studies in rodents suggest that kisspeptin cells may be reprogrammed during development by environmental threats including endocrine disruptors, diet, and stress, with long-term often deleterious effects on reproductive function. Therefore, it remains important to decipher for each species the critical periods of plasticity of the kisspeptin–GPR54 system and to better understand the molecular and cellular mechanisms involved in its developmental programing. Most recently, it has been shown in rats and humans that kisspeptins are already synthesized in some ARC cells well before birth. The physiological significance of these observations have yet to be revealed but one likely hypothesis is that kisspeptins already regulate tonic GnRH release prenatally, hence contributing to early maturation processes of the gonads and possibly to sexual differentiation of some brain circuits. The embryonic period of progenitor cell proliferation and neurogenesis has recently been identified for the ARC kisspeptin neurons of rat. This represents an important first step in the exploration of the morphogenetic processes shaping this neuronal system during early development. In a translational perspective, these studies should help the development of predictive cellular models for assessing the danger of environmental chemicals on reproductive function.

## Conflict of Interest Statement

The authors declare that the research was conducted in the absence of any commercial or financial relationships that could be construed as a potential conflict of interest.
